# Fat-to-muscle Ratio: A New Indicator for Coronary Artery Disease in Healthy Adults

**DOI:** 10.7150/ijms.62871

**Published:** 2021-10-02

**Authors:** Youngmi Eun, Su Nam Lee, Sang-Wook Song, Ha-Na Kim, Se-Hong Kim, Yun-Ah Lee, Sung-Goo Kang, Jun-Seung Rho, Ki-Dong Yoo

**Affiliations:** 1 Department of Family Medicine, Yeouido St. Mary's Hospital, College of Medicine, The Catholic University of Korea, Seoul, Republic of Korea.; 2 Division of Cardiology, Department of Internal Medicine, St. Vincent's Hospital, College of Medicine, The Catholic University of Korea, Suwon-si, Republic of Korea.; 3 Department of Family Medicine, St. Vincent's Hospital, College of Medicine, The Catholic University of Korea, Suwon-si, Republic of Korea.

**Keywords:** Skeletal muscle, body fat, coronary artery disease

## Abstract

**Background:** Coronary artery disease (CAD) is an important issue in public health. Previous studies have shown that the ratio of fat to muscle mass is a significant predictor of metabolic disease, and it is known to be associated with atherosclerosis. In this study, we evaluated the association between the fat-to-muscle ratio (FMR) and CAD in healthy adults.

**Methods:** A total of 617 participants without diabetes mellitus, hypertension, known CAD, or stroke who visited the Health Promotion Center from 2009 to 2018 were included in this study. Computed tomography imaging and bioelectrical impedance analysis were used to ascertain the coronary artery calcium (CAC) score, degree of CAD, and FMR.

**Results:** Univariate logistic regression analysis showed that old age, male sex, smoking history, creatinine, aspartate aminotransferase, gamma-glutamyl transferase, uric acid, total cholesterol, and low-density lipoprotein cholesterol were significantly associated with CAC. After adjusting for potential confounding covariates, the presence of CAC was independently associated with FMR (OR, 1.014; 95% CI, 1.002-1.026; p = 0.019. The association was maintained even after adjusting for body mass index and waist circumference (odds ratio, 1.019; 95% confidence interval, 1.004 -1.034; *P* = 0.012).

**Conclusion:** In this study, a high FMR was significantly associated with CAC. A large-scale prospective study on the association with FMR and cardiovascular diseases is necessary to confirm this relationship.

## Introduction

Coronary artery disease (CAD) is a serious health problem. According to a World Health Organization report, ischemic heart disease was the most common cause of death in 2016, with 15.2 million cases recorded 1. Due to the adoption of an increasingly Westernized lifestyle, the CAD mortality rate in Korea is also increasing 2. Because heart disease can cause serious economic and medical burdens, prevention and early diagnosis of heart disease is important for public health 3.

Fat and muscle are important body tissues for metabolic regulation 4, 5, and it is known that an imbalance between fat and muscle is associated with atherosclerosis and cardiovascular disease 6, 7. The World Health Organization has defined overweightness and obesity as conditions of “abnormal or excessive fat accumulation” that present a risk to health 8. Obesity is a major cause of CAD 9; however, the general definition of obesity using body mass index (BMI) has limitations in that it does not sufficiently reflect the subject's body composition 10. Body composition could be measured by dual-energy X-ray absorptiometry or computed tomography (CT), which is more accurate methods; however, these methods are more expansive and come with a degree of radiation exposure. As such, to compensate for the known limitations, several parameters related to body composition have been proposed to replace BMI 11-15.

Recently, a Chinese study reported that the fat-to-muscle ratio (FMR), which compares the amounts of fat and muscle in the body, is significantly associated with metabolic syndrome 15. There have been reports of a relationship between FMR and fatty liver, diabetes, and other metabolic diseases 13, 14, 16, 17. FMR is a noninvasive and convenient indicator for metabolic abnormalities, and the confirmation of a relationship between cardiovascular disease and FMR is expected to increase the latter's use in medicine. However, not much has been reported regarding the relevance of FMR and coronary atherosclerotic diseases. Therefore, the present study aimed to examine the relationship between FMR and CAD in healthy adults using bioelectrical impedance analysis (BIA) and coronary CT angiography.

## Materials and Methods

### Study population

**Figure 1:**
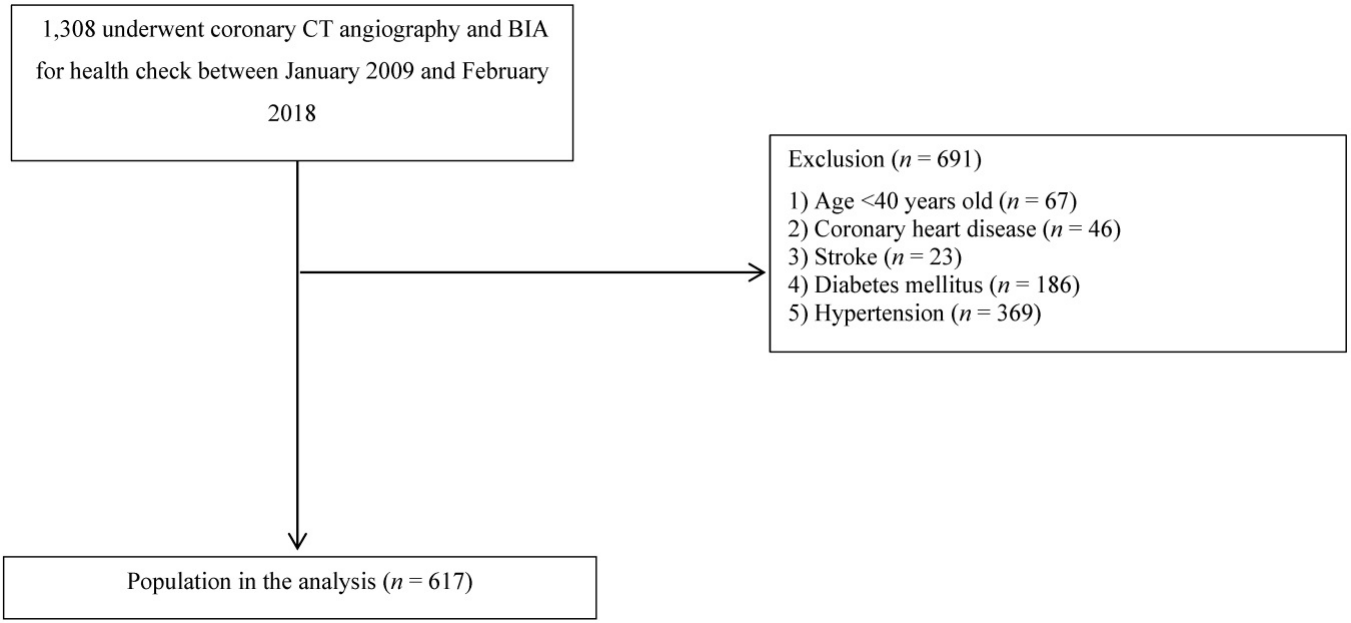
A flowchart of the study population selection process.

From January 2009 to February 2018, a total of 1,308 patients undergoing coronary CT angiography and BIA who visited the Health Promotion Center of St. Vincent's Hospital, Catholic University of Korea, Seoul, Republic of Korea, were enrolled in this retrospective, observational study. Patients younger than 40 years of age (n = 67) or those with CAD (n = 46), stroke (n = 23), diabetes mellitus (DM) (n = 186), or hypertension (HTN) (n = 369) were excluded (Figure 1). Subjects with a systolic blood pressure of at least 140 mmHg, a diastolic blood pressure of at least 90 mmHg, or a history of antihypertensive drug use were defined as having HTN. Subjects with a fasting serum glucose level of at least 126 mg/dL, a glycated hemoglobin concentration of at least 6.5%, or a history of hypoglycemic agent use were defined as having DM. A total of 617 patients were finally included in this study, and this study was exempted from the need to gather written informed consent from participants because all medical data were reviewed retrospectively. For the purpose of this study, all data records were anonymously identified and analyzed. Finally, this study was approved by the institutional review board of St. Vincent's Hospital at the Catholic University of Korea (approval no. VC20RISI0142).

### Anthropometric and laboratory measurements

Anthropometric measurements were performed in duplicate, and the results of such were averaged by trained examiners. Height and weight were measured after an overnight fast, and BMI was calculated as each participant's weight in kilograms divided by the square of their height in meters. Blood pressure measurements were collected in the sitting position after a period of rest for at least 10 minutes. Waist circumference was measured at the midpoint between the lowest rib and the anterior iliac crest in the standing position. All subjects completed a questionnaire that solicited information regarding their age, smoking history, and medical history. A smoking history was defined as being either a current smoker or an ex-smoker. A history of dyslipidemia was defined as a previous diagnosis of such made before the current examination and the use of lipid-lowering agents.

After 12 hours of fasting, blood samples were collected from the antecubital vein of each participant. Laboratory data included the levels of total cholesterol (TC), triglycerides, low-density liporotein cholesterol (LDL-C), high-density lipoprotein cholesterol, fasting serum glucose, glycated hemoglobin (HbA1c), aspartate aminotransferase (AST), alanine aminotransferase, gamma-glutamyl transferase (GTP), uric acid, C-reactive protein, and creatinine.

### Assessment of body fat composition

Body composition measurements, including skeletal muscle mass (kg), body fat mass (kg), waist-to-hip ratio (WHR), and visceral fat area (VFA), were estimated using a multifrequency BIA (InBody 720; Biospace Co., Seoul, Korea). When measuring the body weight, fat mass, and muscle mass, participants were asked to wear light clothing and stand barefoot. FMR was calculated by dividing the body fat mass (kg) by the skeletal muscle mass (kg).

### Assessment of the coronary arteries

To assess coronary arteries, 64-slice multidetector CT scanners (Sensation 64; Siemens, Erlangen, Germany) were used. All subjects fasted for at least six hours prior to examination, and those with heart rates of more than 65 beats/min received a β-blocker to lower their heart rate for testing. Subjects received one 60- to 70-mL dose of nonionic contrast (Ultavist 370; Schering, Berlin, Germany) by injection, followed by 40 mL of saline. CAD was observed using coronary artery calcium score (CACS), high coronary artery calcium (CAC), atheroma, and obstructive CAD (OCAD). The CACS was measured based on the standards of the Agatston score, and CAC was considered present if the CACS was greater than zero points. In addition, a high CACS was defined as an Agatston score of greater than 100 points. We defined the presence of atheroma in the presence of coronary artery stenosis or plague on CT. A diagnosis of OCAD was made based on maximal intraluminal stenosis in all segments of the major epicardial coronary artery at a 50% stenosis threshold.

### Statistical Analysis

Continuous variables are expressed as the mean ± standard deviation values and were compared using an independent t-test. Categorical variables were presented as total numbers and percentages and were compared using the chi-squared test. Logistic regression analysis was performed to determine independent predictors associated with coronary artery atherosclerosis. Variables with significant associations during univariate analysis were entered into the multivariate analysis. Odds ratios (ORs) and 95% confidence intervals (CIs) were also calculated. A* p*-value of less than 0.05 was considered to be statistically significant. A receiver-operating characteristic (ROC) curve analysis was performed for the area under the ROC curve (AUC), and 95% CIs, sensitivity, and specificity were used to assess the cutoff values of FMR for the prediction of CAD. All analyses were performed using SAS version 9.4 (SAS Institute, Cary, NC, USA).

## Results

### Participant characteristics

A total of 617 participants (including 316 men and 301 women) without DM, HBP, known CAD, or stroke were analyzed in this study. Detailed information about the participants is provided in Table 1. The proportion of participants with CAC was 22.0% (n = 136). The participants with a CACS of more than zero points were older, more likely to have a history of smoking, and to be male, with higher concentrations of creatinine, AST, GTP, uric acid, TC, and LDL-C (p <0 .05). Skeletal muscle, WHR, and VFA were significantly higher among participants with a CACS of more than zero points than those with a CACS of zero points. FMR was significantly lower in participants with a CACS of more than zero points than in participants with a CACS of zero points.

### Factors associated with coronary artery disease

In the univariate logistic regression (Table 2), age, male sex, and smoking were significantly associated with a CACS of more than zero points. In laboratory tests, elevations of serum TC, LDL-C, creatinine, AST, GTP, and uric acid were associated with the presence of coronary calcium. Table 3 shows the multivariate analysis results of FMR and CAD-associated factors observed by adjusting significant indicators in the univariate logistic regression analysis. FMR showed a negative relationship with a CACS of more than zero points and atheroma in the uncorrected model but a positive relationship after adjusting for age and sex. CACS had a significant relationship in the overall model. In model 2, after adjusting for potential confounding covariates, the presence of CAC was independently associated with FMR (OR, 1.014; 95% CI, 1.002-1.026; p = 0.019). Furthermore, the association of FMR and CAC was maintained even after adjusting for BMI and waist circumference (OR, 1.019; 95% CI, 1.004-1.034; p= 0.012).

**Figure 2:**
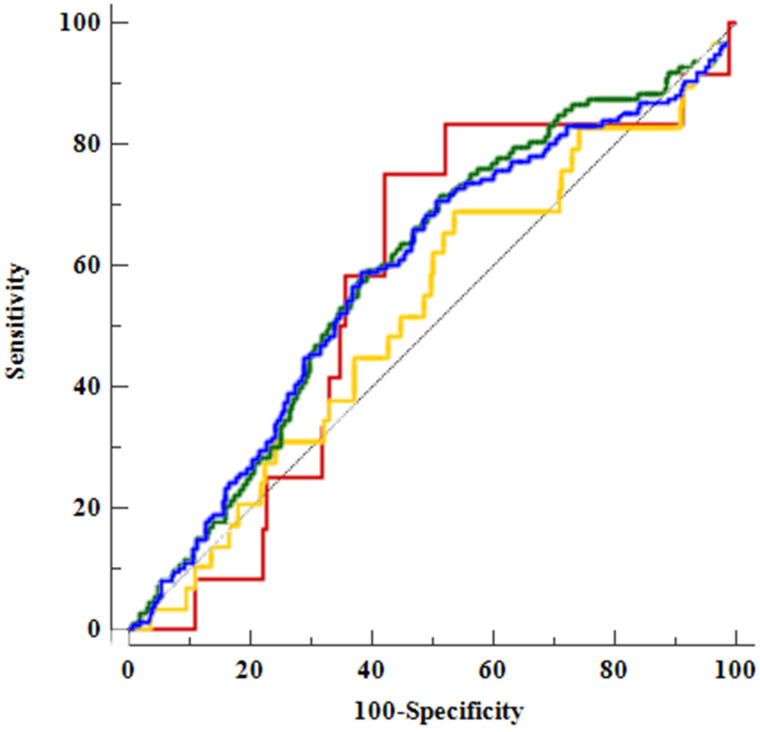
Comparison of areas under the receiver operating characteristic (ROC) curve (AUCs) for the fat-to-muscle ratio and among the factors associated with coronary artery disease using ROC curves. The AUC for CACS, 0.59 (95% confidence interval [CI], 0.55-0.63), is denoted by a blue line; that for high CACS, 0.52 (95% CI, 0.48-0.56), is denoted by a yellow line; that for atheroma, 0.60 (95% CI, 0.56-0.64), is denoted by a green line; and that for OCAD, 0.57 (95% CI, 0.53-0.61), is denoted by a red line.

In ROC analysis of body composition factors and CAD (Table 4 and Figure 2), FMR did not demonstrate a clear predictive power for CAD but did exhibit significant results for a CACS of more than zero points and atheroma.

## Discussion

In this study, we evaluated the relationship between FMR, and CAD associating factors, including CAC. A higher FMR was significantly associated with CAC in healthy adults, even though the association was maintained after adjusting for BMI and waist circumference.

FMR is known to be a strong predictive tool for metabolic syndrome in studies conducted in China and Korea 13, 15. In addition, a Colombian research group demonstrated good discriminatory power of FMR for detecting metabolic syndrome, even in young adults 18. The association between FMR and metabolic diseases has been confirmed in several studies. Gamboa-Gomez et al. 14 demonstrated a strong association between FMR and glucose metabolic disorder, and a Taiwanese group showed that FMR is strongly correlated with DM, HTN, and pre-DM 16.

In accordance with our study, Chen et al. suggested that the assessment of FMR while incorporating metabolic syndrome had a better predictive ability for discerning the CAD risk than other definitions 16. However, not enough studies exist that focus on FMR and coronary atherosclerosis. To the best of our knowledge, our study is the first investigation to compare FMR and CAC. Currently, the mechanisms for the association between FMR and coronary atherosclerosis are unknown; however, it has been reported that an imbalance between muscle and fat masses is associated with chronic inflammation, and fat mass actively secretes pro-inflammatory adipocytokines, which have potentially direct catabolic effects on muscle 19. In addition, increased plasma fatty acids promote the accumulation of fat in the muscles, leading to abnormalities in mitochondrial function and resulting in muscle insulin resistance 20. These cycles between muscle loss and fat gain lead to sarcopenia, additional fat accumulation, and metabolic derangement. Therefore, the relationship between fat and muscle may affect the association between FMR and CAD.

Although a relationship between FMR and CAC was observed, it was also found that the predictive power of CAC was weak in the context of FMR. This result seems to be because FMR does not reflect the distribution or type of fat or the quality of muscle. Previous studies have shown that subcutaneous adipose tissue 21 or lower body fat 22 has a negative relationship with all-cause death or cardiovascular disease risk. Furthermore, intramuscular adipose tissue or lower quality muscle showed positive associations with CAC 23, 24. Despite these limitations, however, FMR is a convenient and economical indicator related to metabolic diseases and CAC. Therefore, it is suggested that additional prospective studies are warranted to confirm this relationship.

This study increases our understanding of the association between FMR and CAD using CAC in healthy adults. However, it has some limitations. First, this study was a cross-sectional investigation and it is difficult to characterize the causal relationship as a result. Second, because this study involved those who wanted to undergo coronary artery CT imaging as part of their health checkup, the number of study participants was small and the study time was long, and this could have resulted in bias in the study. Third, angiography was not performed to confirm the degree of arteriosclerosis, and CAC was not classified or compared according to severity. Therefore, it is considered that related considerations should be taken into account when conducting large-scale studies in the future.

## Conclusions

Our results showed that a high FMR was significantly associated with CAC. This association persisted even after adjusting for covariates, including BMI and waist circumference. This result may support FMR as a predictive marker of early coronary atherosclerosis, although further large-scale prospective studies on the relationship of FMR with coronary atherosclerosis are necessary to validate the former as an indicator of CAD.

## Figures and Tables

**Table 1:** Baseline characteristics of participants (n = 617)

Variable	All	CACS=0 (n=481)	CACS>0 (n=136)	P value
Age (years)	55.9 ± 7.7	54.8 ± 7.1	59.9 ± 8.4	< 0.001
Men	316 (51.2%)	215(44.7%)	101(74.3%)	< 0.001
BMI (kg/m^2^)	23.9 ± 2.6	23.8± 2.6	24.2 ± 2.8	0.109
Smoking	259 (42.0%)	178 (37.0%)	81 (59.6%)	< 0.001
Waist circumference (cm)	84.0 ± 8.1	83.7 ± 8.0	84.9 ± 8.2	0.142
Dyslipidemia	38 (6.2%)	25 (5.2%)	13 (9.6%)	0.062
SBP (mmHg)	119.7 ± 11.1	119.3 ± 11.2	121.2 ± 10.3	0.079
DBP (mmHg)	72.9 ± 7.9	73.0± 8.0	72.5 ± 7.4	0.564
Fasting glucose (mg/dL)	93.4 ± 9.5	93.0± 9.1	94.6 ± 10.5	0.117
HbA1C (%)	5.60 ± 0.35	5.59 ± 0.36	5.62± 0.31	0.393
TC (mg/dL)	205.8 ± 34.7	204.2 ± 34.2	211.6 ± 36.2	0.029
HDL-C (mg/dL)	49.1 ± 11.5	49.3± 11.6	48.4 ± 11.2	0.436
LDL-C (mg/dL)	132.6 ± 31.2	130.7 ± 30.8	139.4 ± 31.6	0.004
Triglyceride (mg/dL)	119.4 ± 70.8	117.2 ± 71.8	127.1 ± 66.7	0.152
Creatinine (mg/dL)	0.78 ± 0.16	0.77 ± 0.15	0.82 ± 0.16	0.001
AST (IU/L)	23.4 ± 8.9	22.9 ± 8.4	25.1 ± 10.4	0.023
ALT (IU/L)	24.1 ± 14.0	23.6 ± 14.0	25.8 ± 14.0	0.095
GTP (IU/L)	31.6 ± 35.7	29.9 ± 35.8	37.6 ± 35.1	0.026
Uric acid (mg/dL)	5.31 ± 1.33	5.22 ± 1.31	5.63 ± 1.38	0.002
CRP (mg/dL)	0.17 ± 0.47	0.16 ± 0.38	0.21 ± 0.71	0.207
Body fat (kg)	18.0 ± 5.0	18.0± 4.8	17.7 ± 5.6	0.597
Skeletal muscle (kg)	25.5 ± 5.7	25.2 ± 5.8	26.7 ± 5.3	0.005
WHR	0.90 ± 0.05	0.90 ± 0.04	0.92 ± 0.05	< 0.001
VFA (cm^2^)	101.8 ± 28.2	99.9 ± 26.2	108.6 ± 33.6	0.006
FMR (kg/kg)	0.74 ± 0.27	0.76 ± 0.27	0.69 ± 0.28	0.019

***Abbreviations*:** CACS, coronary artery calcium score; BMI, body mass index; SBP, systolic blood pressure; DBP, diastolic blood pressure; HbA1c, glycated hemoglobin; TC, total cholesterol; HDL-C, high-density lipoprotein cholesterol; LDL-C, low-density lipoprotein cholesterol; AST, aspartate aminotransferase; ALT, alanine aminotransferase; GTP, gamma-glutamyl transferase; CRP, C-reactive protein; WHR, waist-to-hip ratio; VFA, visceral fat area; FMR, fat-to-muscle ratio. Data are presented as the mean ± standard error values for continuous variables or as percentages for categorical variables.

**Table 2:** Factors associated with coronary artery calcium

	OR	95% CI		p-value
		lower	upper	
Age (years)	1.092	1.064	1.122	<0.001
Male (%)	3.570	2.335	5.457	<0.001
Body mass index (kg/m^2^)	1.061	0.987	1.140	0.109
Smoking	2.507	1.699	3.700	<0.001
Waist circumference (cm)	1.018	0.994	1.043	0.142
Dyslipidemia	1.928	0.958	3.879	0.066
SBP (mmHg)	1.016	0.998	1.034	0.079
DBP (mmHg)	0.993	0.969	1.017	0.563
Fasting glucose (mg/dL)	1.017	0.997	1.038	0.089
HbA1C (%)	1.151	0.660	2.008	0.619
TC (mg/dL)	1.006	1.001	1.012	0.030
HDL-C (mg/dL)	0.993	0.977	1.010	0.435
LDL-C (mg/dL)	1.009	1.003	1.015	0.005
Triglyceride (mg/dL)	1.002	0.999	1.004	0.157
Creatinine (mg/dL)	7.657	2.269	25.834	0.001
AST (IU/L)	1.025	1.005	1.045	0.013
ALT (IU/L)	1.011	0.998	1.024	0.100
GTP (IU/L)	1.005	1.000	1.010	0.035
Uric acid (mg/dL)	1.234	1.073	1.418	0.003
CRP (mg/dL)	1.202	0.830	1.741	0.330

***Abbreviations:*** OR, odds ratio; CI, confidence interval; SBP, systolic blood pressure; DBP, diastolic blood pressure; HbA1c, glycated hemoglobin; TC, total cholesterol; HDL-C, high-density lipoprotein cholesterol; LDL-C, low-density lipoprotein cholesterol; AST, aspartate aminotransferase; ALT, alanine aminotransferase; GTP, gamma-glutamyl transferase; CRP, C-reactive protein.

**Table 3:** Multivariate logistic analysis of coronary artery disease with fat-to-muscle ratio

	low FMR	high FMR	p-value
**CACS>0**			
Crude	1	0.991 (0.984-0.999)	0.019
Model 1	1	1.015 (1.004-1.026)	0.009
Model 2	1	1.014 (1.002-1.026)	0.019
Model 3	1	1.019 (1.004-1.034)	0.012
**High CACS**			
Crude	1	0.998 (0.985-1.012)	0.809
Model 1	1	1.021(1.001-1.042)	0.045
Model 2	1	1.019 (0.997-1.041)	0.091
Model 3	1	1.023 (0.994-1.052)	0.119
**Atheroma**			
Crude	1	0.989 (0.981-0.997)	0.006
Model 1	1	1.005 (0.993-1.016)	0.421
Model 2	1	1.003 (0.991-1.015)	0.667
Model 3	1	1.004 (0.988-1.019)	0.646
**OCAS**			
Crude	1	0.994 (0.972-1.016)	0.581
Model 1	1	1.004 (0.975-1.035)	0.770
0Model 2	1	1.005 (0.974-1.036)	0.773
Model 3	1	0.970 (0.925-1.017)	0.207

***Abbreviations:*** FMR, fat-to-muscle ratio; CACS, coronary artery calcium score; OCAS, obstructive coronary artery disease. Data are presented as the odds ratio (95% confidence interval) values. Model 1: adjusted for age and sex. Model 2: adjusted for age, sex, smoking, creatinine, AST, GTP, uric acid, TC, and LDL-C. Model 3: adjusted for age, sex, smoking, creatinine, AST, GTP, uric acid, TC, LDL-C, body mass index, and waist circumference.

**Table 4:** Receiver-operating characteristic curve analysis of the fat-to-muscle ratio for the diagnostic prediction of coronary artery disease

	cut-off point	Sensitivity	Specificity	PPV	NPV	AUC (95% CI)	P value
CACS	0.650	0.588	0.615	0.302	0.841	0.586 (0.546-0.626)	0.002
High CACS	0.738	0.690	0.463	0.060	0.968	0.523 (0.483-0.563)	0.667
Atheroma	0.745	0.717	0.486	0.238	0.885	0.596 (0.556-0.635)	<0.001
OCAD	0.650	0.750	0.577	0.034	0.992	0.568 (0.528-0.607)	0.364

***Abbreviations:*** PPV, positive predictive value; NPV, negative predictive value; AUC, area under the receiver-operating characteristic curve; CI, confidence interval; CACS, coronary artery calcium score; OCAS, obstructive coronary artery disease.
